# Auxin-Responsive DR5 Promoter Coupled with Transport Assays Suggest Separate but Linked Routes of Auxin Transport during Woody Stem Development in *Populus*

**DOI:** 10.1371/journal.pone.0072499

**Published:** 2013-08-15

**Authors:** Rachel Spicer, Tracy Tisdale-Orr, Christian Talavera

**Affiliations:** 1 Department of Botany, Connecticut College, New London, Connecticut, United States of America; 2 Rowland Institute at Harvard, Cambridge, Massachusetts, United States of America; 3 Eurofins Eaton Analytical, Inc., Gas Chromatography Department, Monrovia, California, United States of America; University of Nottingham, United Kingdom

## Abstract

Polar auxin transport (PAT) is a major determinant of plant morphology and internal anatomy with important roles in vascular patterning, tropic growth responses, apical dominance and phyllotactic arrangement. Woody plants present a highly complex system of vascular development in which isolated bundles of xylem and phloem gradually unite to form concentric rings of conductive tissue. We generated several transgenic lines of hybrid poplar (*Populus tremula* x *alba*) with the auxin-responsive DR5 promoter driving GUS expression in order to visualize an auxin response during the establishment of secondary growth. Distinct GUS expression in the cambial zone and developing xylem-side derivatives supports the current view of this tissue as a major stream of basipetal PAT. However, we also found novel sites of GUS expression in the primary xylem parenchyma lining the outer perimeter of the pith. Strands of primary xylem parenchyma depart the stem as a leaf trace, and showed GUS expression as long as the leaves to which they were connected remained attached (i.e., until just prior to leaf abscission). Tissue composed of primary xylem parenchyma strands contained measurable levels of free indole-3-acetic acid (IAA) and showed basipetal transport of radiolabeled auxin (^3^H-IAA) that was both significantly faster than diffusion and highly sensitive to the PAT inhibitor NPA. Radiolabeled auxin was also able to move between the primary xylem parenchyma in the interior of the stem and the basipetal stream in the cambial zone, an exchange that was likely mediated by ray parenchyma cells. Our results suggest that (a) channeling of leaf-derived IAA first delineates isolated strands of pre-procambial tissue but then later shifts to include basipetal transport through the rapidly expanding xylem elements, and (b) the transition from primary to secondary vascular development is gradual, with an auxin response preceding the appearance of a unified and radially-organized vascular cambium.

## Introduction

The plant hormone auxin serves as a major regulator of plant morphology and anatomy with critical roles in developmental processes including embryogenesis, phyllotactic and vascular patterning, apical dominance and tropic responses. The predominant form of auxin in plants is indole-3-acetic acid (IAA), a small molecule that effects changes in gene expression by targeting transcriptional repressors for degradation [1]. That a single molecule can elicit such a diverse array of developmental responses is a function of its precise localization, where its effect depends on the genetic background of the cells in which it is acting. Dynamic localization of IAA is achieved through a highly regulated and directional cell-cell transport termed polar auxin transport (PAT). One of the best-studied and most dramatic examples of PAT in plant development occurs during vascular patterning. In leaf primordia, the basipetal channeling (canalization hereafter) of IAA from a convergence point in the epidermis down through a narrow file of cells in the center of the emerging primordium determines the location of procambium (and what will ultimately become the midvein), the meristematic tissue from which all primary xylem and phloem is produced [2]. Similarly, acropetal flow of auxin toward the root tip determines the location of procambium and hence the primary vasculature of the stele [3]. Although originally conceived through classical development studies, the canalization hypothesis [4], [5] has been repeatedly supported and refined by molecular work demonstrating that auxin transport and accumulation is mediated by at least three classes of specific membrane proteins (PIN, AUX1/LAX and MDR/ABCB families; for reviews see [6], [7], [8]). The PIN proteins in particular appear to satisfy several critical requirements of the canalization hypothesis: they are often asymmetrically localized in the plasma membrane where they are able to be rapidly repositioned (e.g., [9]) and their localization is auxin-responsive, thus providing the positive feedback mechanism required to effect 'canalized' flow [10].

Our understanding of the role of PAT in vascular patterning is based almost entirely on herbaceous plants in which vascular development is limited to the production of primary xylem and phloem from one or more procambial strands. In contrast, in woody plants these isolated strands (or in some cases continuous rings) of procambium grade developmentally into true cambium [11], [12], the lateral meristem responsible for thickening axes through the production of secondary xylem and phloem. In most woody plants the vascular cambium exists as a single, continuous ring of meristematic initials that undergo periclinal divisions to produce radial files of vascular tissue [13]. Less frequent are anticlinal divisions, which add the new initials required to maintain cambial continuity and keep pace with the expanding circumference of the stem. Auxin transport is logically implicated in the development of secondary vascular tissues as well, but its role there is poorly understood, in large part due to the methodological challenges and long time scales required when working with woody plants. Given the importance of plant secondary growth to the fields of materials science, forestry and bioenergy production, there is a clear need for a better understanding of this process.

The cambial zone − a region that includes both the cambial initials and their immediate derivatives (i.e., phloem and xylem mother cells) − has long been known to contain a high concentration of auxin [14], [15] and all experimental evidence suggests that this auxin is transported basipetally in the stem [16−21]. Accurate quantitation of free auxin (i.e., IAA not conjugated to any other moiety) at high spatial resolution through the cambial zone of *Pinus* and *Populus* shows a peak concentration of IAA in the vicinity of the vascular cambium that rapidly declines through the zones of cell differentiation and expansion, reaching undetectable amounts in mature xylem and phloem [22−26]. Similarly, genes coding for members of the PIN, AUX1/LAX and ABCB families of auxin transport proteins are expressed in the cambial zone and developing secondary tissues at levels that vary with radial and axial position in the stem (i.e., with developmental stage) and with seasonal shifts between active growth and dormancy [27−30]. This latter finding provides mechanistic support for the physiological observation that auxin transport in woody stems is greatly reduced during dormancy, despite only modest reductions in IAA concentration in stem tissue [25−28], . The potential for feedback between IAA concentration and transport is also suggested, as several members of all three families of transporters show increased expression in response to exogenous IAA in developing stems of *Populus* [27], [30], [32]. Finally, both modeling and experimental work suggest that basipetal auxin transport in the cambial zone determines the grain angle in developing xylem, a property that is likely a function of both cell polarity and canalization [18], [33].

Although the role of auxin signaling in secondary growth and development is certain to be complex, the woody stem is viewed as a relatively simple system in terms of PAT, as it is often stated that PAT is restricted to the cambial zone and that little or no biosynthesis or (de)conjugation occurs in the stem [25], [27]. Two main lines of evidence suggest that a more detailed understanding of auxin transport in woody stems is warranted. First, the developmental origin of leaf/stem vascular connections is quite complex and the addition of secondary growth complicates matters. Although we have good insight into how the basipetal canalization of IAA in leaf primordia leads to procambium formation and in turn, vascular bundle differentiation [2], [34], the ultimate fate of that IAA and how new bundles unite with existing vasculature is poorly understood. During primary growth, xylem and phloem remain together in the bundles of both leaf and stem, but they become physically separated in the stem by the action of the vascular cambium such that each bundle is effectively split during secondary growth: primary xylem is gradually embedded in secondary xylem such that it traverses the stem from the outer edge of the pith to the departing leaf trace, while primary phloem remains continuous along the outside of the stem [11], [12]. The narrow strips of procambium between primary xylem and phloem in the bundle grade into a continuous cambium below [35], but it is not known if this represents a continuous route for PAT down the stem. Second, there is increasing evidence that auxin biosynthesis may occur throughout the plant body rather than being restricted to young shoot apices [36−38], and that mature leaves may contribute to the basipetal, polar auxin stream in stems via export from the phloem [39], [40]. Indeed, although non-polar transport of conjugated forms of IAA is likely to occur in secondary phloem [41], [42], the few studies that have quantified conjugates in woody stems have focused on the cambial zone [20], [22], [43], [44].

It is difficult to overstate the functional significance of this developmental process for the transport of water and photosynthate, particularly for woody plants in which the continuity of vascular tissue linking leaf, stem and root changes fundamentally during secondary growth. Here we describe the results of work designed to characterize auxin transport and response at several stages of development of the vascular cambium in *Populus* using an auxin-responsive reporter (specifically, the DR5 promoter driving GUS expression) coupled with radiolabeled auxin transport assays. Our results suggest that in addition to the cambial zone, strands of parenchyma associated with primary xylem serve as route for basipetal PAT in developing woody stems. Capacity for the exchange of radiolabeled auxin between these parenchyma strands and the cambial zone via ray parenchyma further suggests that complex pathways for auxin transport exist in woody stems.

## Materials and Methods

### Plant material and growth conditions

Auxin-responsive *Populus tremula* x *alba* reporter lines were generated by *Agrobacterium*-mediated leaf disk transformation of the hybrid clone INRA 717-1B4 following a protocol modified from [45]. The synthetic DR5 promoter [46] was cloned into the Gateway™ binary vector pKGWFS7 [47], which contains a GFP-GUS fusion as a bifunctional reporter [48]. Co-cultivation of approximately 200 leaf disks with *Agrobacterium tumefaciens* strain GV3101 containing this vector yielded hundreds of micropropagated shoots grown on selective media. Fourteen independent lines (i.e., shoots derived from independent leaf disks) were selected, verified to contain the entire DR5_pro_:GUS-GFP construct, and propagated for further evaluation. We refer to these lines as PtaDR5 plants. Both *wt* (untransformed 717) and PtaDR5 plants were grown *in vitro* and later transferred to soil and grown in a greenhouse. *In vitro* grown plants were maintained at 24°C under 16-hr days (150 µmol m^−2^ s^−1^ light from a combination of cool white and full spectrum fluorescent bulbs). Greenhouse temperatures ranged from 24°C to 30°C and 16-hr days were maintained with supplemental metal halide lamps. Plants grown in soil were fertilized with a water-soluble fertilizer (NPK 17:11:10) bi-weekly and transplanted as needed.

Plants were grown for anywhere from one to six months depending on the experiment. We used a 'leaf plastochron index' system [49] to assure that stems were at the same developmental stage for any given test or manipulation. We defined 'the apex' as the tight cluster of leaves above the first internode that could be clearly identified with the unaided eye. The leaf that subtended this internode (i.e., the first leaf beneath the apex) was approximately 1.5 cm long with the basal one-third of the leaf margin still curled. Under our growing conditions, saplings maintained between 100 to 125 leaves beneath the apex before they began to abscise and had an outer stem diameter of about 1.5 cm at a position 100 nodes beneath the apex.

### Auxin response in PtaDR5 lines

All 14 PtaDR5 lines were tested for an auxin response by incubating plant tissue in half-strength MS liquid growth media (half-strength MS salts, 2% sucrose, 0.25 mg ml^−1^MES, 0.04 mg ml^−1^ glycine, and 0.2 mg ml^−1^ myo-inositol; pH 6.0) containing 30 µM IAA at 22°C for 4−12 hrs following brief vacuum infiltration. Whole *in vitro* grown plantlets and stem and root segments from both *in vitro* and greenhouse grown plants were tested and the auxin response was compared against matched controls incubated in the same media without IAA. In order to test for an auxin response to endogenous IAA, lanolin containing 50 mM NPA (N-1-naphthylphthalamic acid in DMSO) was applied in a 0.5-cm-wide ring around the epidermis of stems 0.4 to 1 cm in diameter, covered with foil for 2 weeks, and harvested above and below the application site. Control plants were treated with DMSO in lanolin. GUS assays were performed on fresh and fixed tissue following Jefferson et al [50] with a 4−8 hr incubation at 37 °C in X-Gluc solution containing 2 mM potassium ferrocyanide and 2 mM potassium ferricyanide. For all tissues examined, ice-cold-acetone-fixed and LN2-plunge-frozen tissue was tested to check for wounding artifacts. Acetone fixation greatly reduced but did not eliminate the signal; LN2 freezing did not reduce the signal relative to fresh tissue but did significantly disrupt soft tissues. Unless otherwise stated, images are from fresh tissue in which localization of GUS was verified with matched LN2-frozen tissue. Tissue was cleared in 70% EtOH to remove chlorophyll.

Endogenous GUS expression was characterized in three PtaDR5 lines in more detail during active growth and the onset of dormancy (minimum of 6 plants each at various ages). Expression of GUS was chosen over GFP as a reporter for all experiments because stem tissues generally needed to be sectioned, fixed and cleared, whereas viewing GFP requires live whole mounts. The GFP signal was also weak relative to the background autofluorescence typical of secondary vascular tissue. Dormancy was induced over 12 weeks following transfer to soil. Plants were grown under 8 hr days at 15 °C for four weeks, then 10 °C for eight weeks, at which time plants had set bud and dropped all of their leaves. Dormant plants were compared against actively growing plants with a comparable number of internodes. In both cases fresh tissue was incubated in X-Gluc as described above, fixed in 4% paraformaldehyde (PO_4_-buffered; pH 7.2) for 24 hrs and cleared in 70% EtOH. Tissue browning due to phenolic oxidation was particularly problematic in dormant apices and required additional clearing in 0.5% sodium hypochlorite, which proved more effective than traditional clearing methods using chloral hydrate [51]. Soft tissues (e.g., apices) were embedded in 5% agarose blocks and sectioned at 50 to 100 µm on a vibratome. Woody stem segments were sectioned at 24 µm on a sliding microtome.

### Auxin transport assays

**Figure 1:**
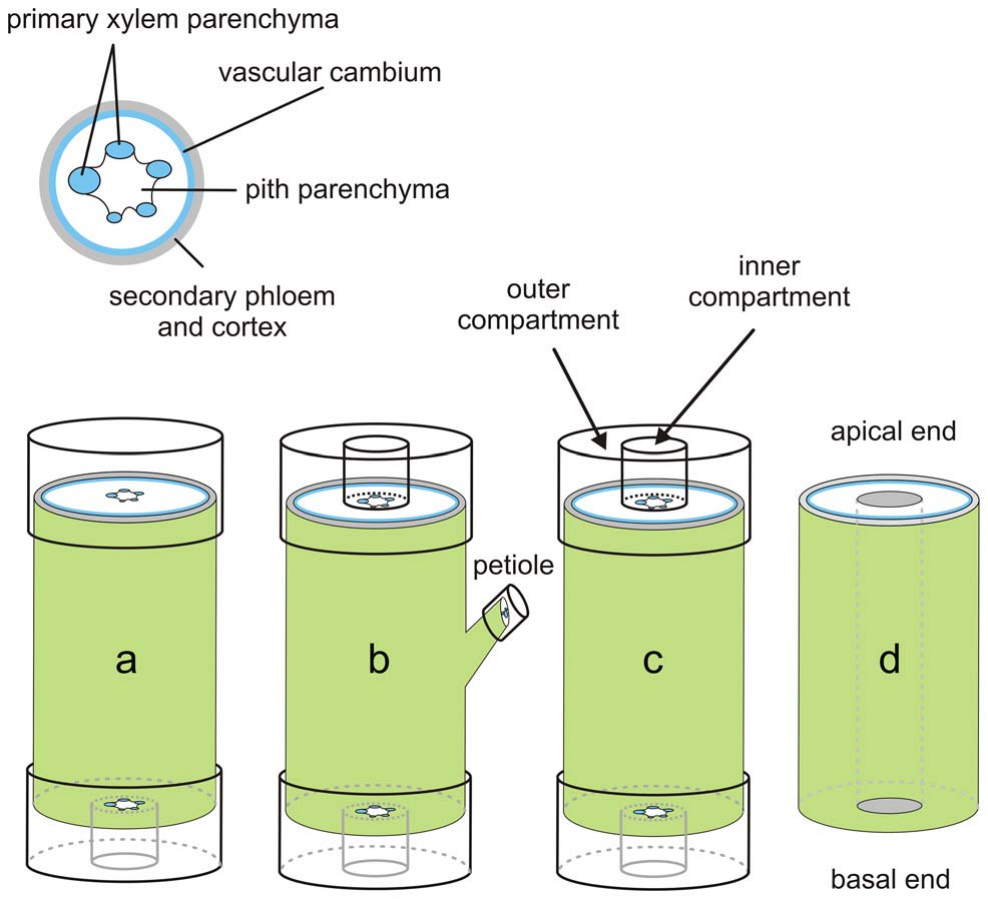
Radiolabeled auxin transport assays. Radiolabeled auxin (^3^H-IAA) or benzoic acid (^3^H-BA) were applied to *Populus* stems (100 nM in 1.5% agar) in a series of transport assays. Inner and outer compartments were created with thin metal rings to separate primary and secondary vascular tissues, respectively. Four separate assays were performed in order to test (a) the capacity of primary versus secondary tissue for basipetal auxin transport, (b) the connection between leaf traces and the two respective compartments, (c) the potential for exchange between primary and secondary tissues, and (d) the potential for outward radial transport of auxin. BA served as a diffusion and non-polar transport control; addition of NPA (10 µM) to the media provided further evidence of polar transport.

The capacity for auxin transport through primary and secondary vascular compartments was measured on stem segments excised from 4- to 6-month-old INRA 717-1B4 trees (untransformed) with radiolabeled IAA in a series of continuous loading assays (Figure 1) based on a modification of Lewis and Muday [52]. Continuous loading assays were performed over pulse chase assays because (a) trimming the ends of woody stems > 1 cm in diameter required the use of a miter saw and was not feasible when working with radioactivity, and (b) relative quantification of radiolabel transport over time was sufficient to demonstrate PAT. Agar (1.25% wt/vol) dissolved in growth media (half-strength MS salts, described above) served as both donor and receiver for radiolabeled compounds. In the first assay, agar containing 100 nM ^3^H-IAA (20 Ci mmol^−1^), 100 nM ^3^H-BA (60 Ci mmol^−1^) or 100 nM ^3^H-IAA + 10 µM NPA was applied to the entire apical end of internodes excised from regions centered 35 and 90 nodes beneath the apex and collected from the inner and outer compartments of the basal end (Figure 1a). The inner compartment was isolated by gently pressing a sharpened brass ring (6 mm diameter x 5 mm height) into the secondary xylem just outside the primary tissue such that it contained the primary xylem poles (i.e., vessels and associated parenchyma) and pith. Foil tape was wrapped around the outside of the stem at both ends to create an outer compartment that included the vascular cambium, secondary xylem and phloem, cortex and epidermis. In a second assay, the connection between mature leaves (about 60 nodes beneath the apex) and stem was tested by applying agar containing each radiolabeled compound (concentrations as above) to a cut petiole in a 0.5 ml eppendorf cap and collecting the agar from both apical and basal, inner and outer compartments (Figure 1b). In a third assay, the potential for exchange between the two compartments was tested by including the radiolabeled compound (concentrations as above) in only one of the two apical compartments (inner or outer) and collecting both basal compartments (Figure 1c). In a final assay, the potential for radial transport in mature stems (about 90 nodes beneath the apex) was measured by drilling a 0.4-cm-diameter hole down the center of a stem segment (Figure 1d), essentially removing the pith, and filling the cavity with lanolin containing one of four radiolabeled compounds (^3^H-IAA, ^3^H-BA, ^3^H-IAA + NPA, or ^3^H-IAA + 10 µM quercetin [a naturally occurring favonol with NPA-like activities; [53]], concentrations as above). Lanolin replaced agar as a delivery medium in this assay because its higher density and lower water content allowed better contact with the surface of the drilled hole. After an 8 hr incubation time, the bark was peeled off and developing xylem was removed with a razor blade and mixed with agitation in 4 mls isopropyl alcohol for 48 hrs. In all transport assays, samples were held upright (apical end up) in humid chambers in the dark at room temperature for 8-16 hours. Agar collected from inner and outer receiver compartments was dissolved in 5% sodium hypochlorite, resuspended in 10 mls of Hionic-Fluor™ scintillation cocktail and counted on a Beckman 6500 LSC. For the radial transport assay, the isopropyl alcohol was resuspended in 8 mls Hionic-Fluor™. The amount of radioactivity recovered from receiver blocks or tissue was normalized for small differences in transport time (owing to the length of time required to harvest samples) and the distance traveled (sample length) such that final units reported are in fmol IAA transported over the time specified.

### IAA quantification

IAA was quantified in primary and secondary tissue by GCMS using ^13^C-IAA as an internal standard [54] following the methods of [55], [56]. Internodal stem segments of 6-month-old, greenhouse-grown INRA 717-1B4 plants were harvested from regions between 40 and 80 nodes beneath the apex from each of five plants. Samples of the same tissue from a single plant were pooled in order to provide sufficient volumes for extraction. Developing secondary xylem was collected by peeling the bark, scraping the surface of the exposed wood with a razor blade and freezing the xylem strips in LN2. Tissue enriched in primary xylem parenchyma was collected by splitting stem segments longitudinally into eight wedges, removing the central pith (the "point" of the wedge) with a razor blade, freezing in LN2 and grating the truncated region up to the secondary xylem using a stainless steel fine-toothed grater. Blocks of mature secondary xylem (excluding any developing or primary xylem) were also frozen in LN2 and grated. In all cases grated tissue was kept frozen on LN2, ground with a mortar and pestle to a fine powder, and weighed into 150 mg aliquots for extraction. ^13^C-IAA was added to each aliquot at 40 ng g^−1^ frozen tissue. Details of the extraction procedure can be found in the above references. Briefly, aliquots were extracted for one hr on ice in 65% isopropanol and 35% 0.2 M imidazole following a 1 min pulsed vortex homogenization. Homogenates were centrifuged, the supernatant diluted 10x in water and applied to a conditioned NH_2_ column. Following a wash series and elution in 0.25% phosphoric acid, the eluate was pH-corrected to 3.2, applied to a conditioned epoxide resin column, washed, and eluted in methanol. The final eluate was methylated with diazomethane, resuspended in ethyl acetate and stored at −80 °C for a maximum of two weeks prior to analysis. Samples were analyzed at the Harvard FAS Center for Systems Biology Mass Spectrometry and Proteomics Resource Laboratory on a Waters Quattro Micro GCMS in SIM mode monitoring ions at m/z 130 and 189 (endogenous IAA, fragment and molecular ions respectively) and 136 and 195 (internal standard, fragment and molecular ions respectively). The GC was equipped with a 30 m fused silica capillary column and 280 °C splitless injector, and the MS ionization (EI) was set to 70 eV.

### Statistical analyses

Results of auxin transport assays were compared with paired t-tests to account for the lack of independence between inner and outer compartments within the same aged segments, and between different aged segments within the same plant. Only *a priori* comparisons of interest were tested, namely the effects of PAT inhibitors (e.g., the effect of NPA inclusion on IAA transport within a particular longitudinal compartment, and the effect of NPA and quercetin on radial transport on segments from the same plant). Results from the diffusion control (^3^H-BA transport) are shown for comparison but statistical tests comparing BA and IAA transport were only conducted for radial transport assays, where BA movement was likely via symplasmic transport through rays and not strictly via intercellular diffusion.

## Results

### PtaDR5 response to exogenous and endogenous auxin

**Figure 2:**
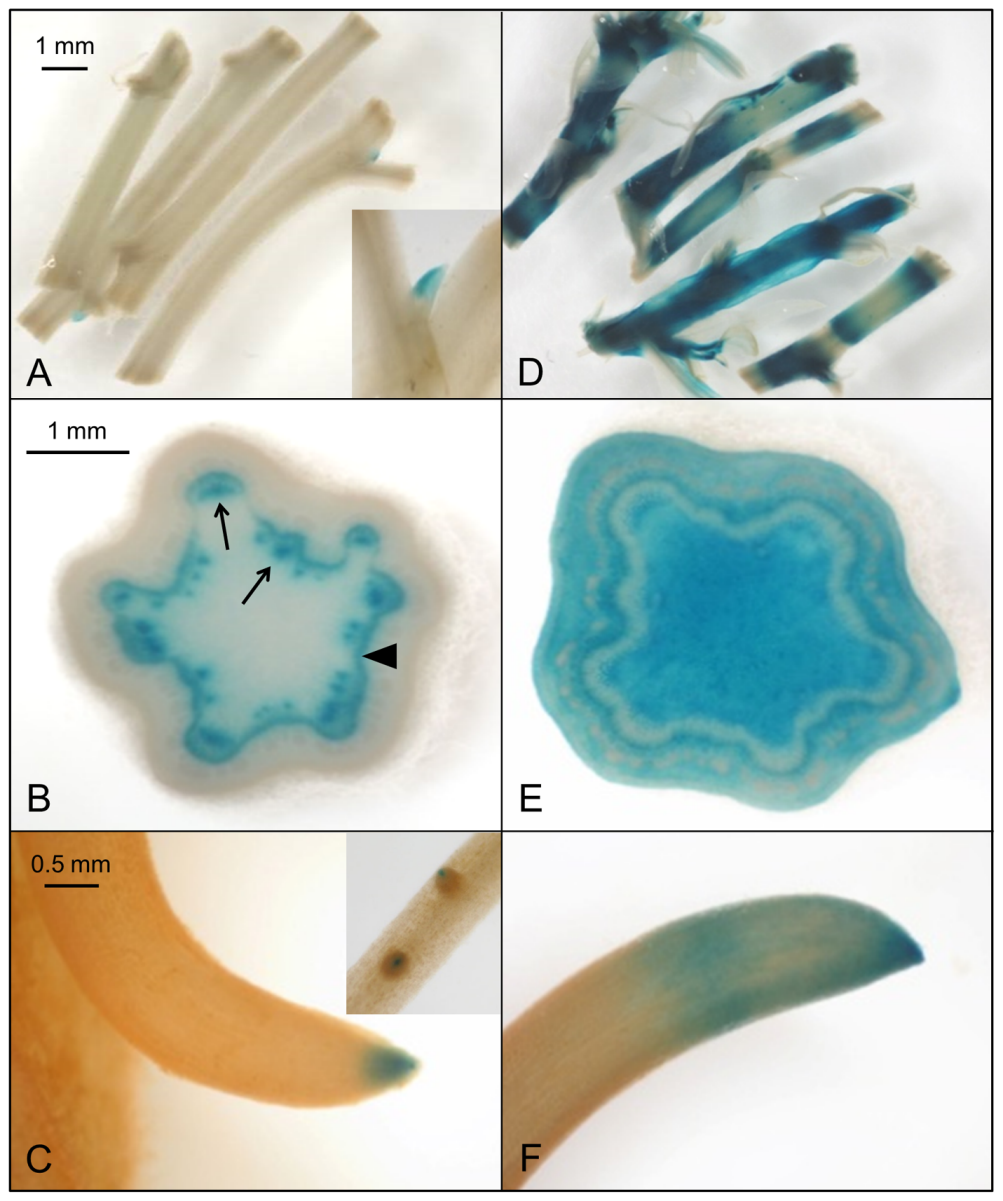
GUS expression in PtaDR5 tissue indicates an auxin response. GUS expression in a representative PtaDR5 line (a, b, c; CONTROL showing endogenous expression) and following incubation in 30 µM IAA (d, e, f; TREATED showing response to exogenous IAA). Stem segments (a−e) and roots (c and f) from plants grown *in vitro* were incubated in 1/2 strength MS growth media with or without IAA for 12 hours in the dark prior to histochemical staining with X-Gluc. The strong response to exogenous auxin (d, e, f) and pattern of endogenous GUS expression in axillary buds (a), cambial zone and primary xylem parenchyma (b; arrowhead and arrows, respectively), and primary and lateral root tips (c and inset, respectively) were consistent with other independently transformed PtaDR5 lines.

**Figure 3:**
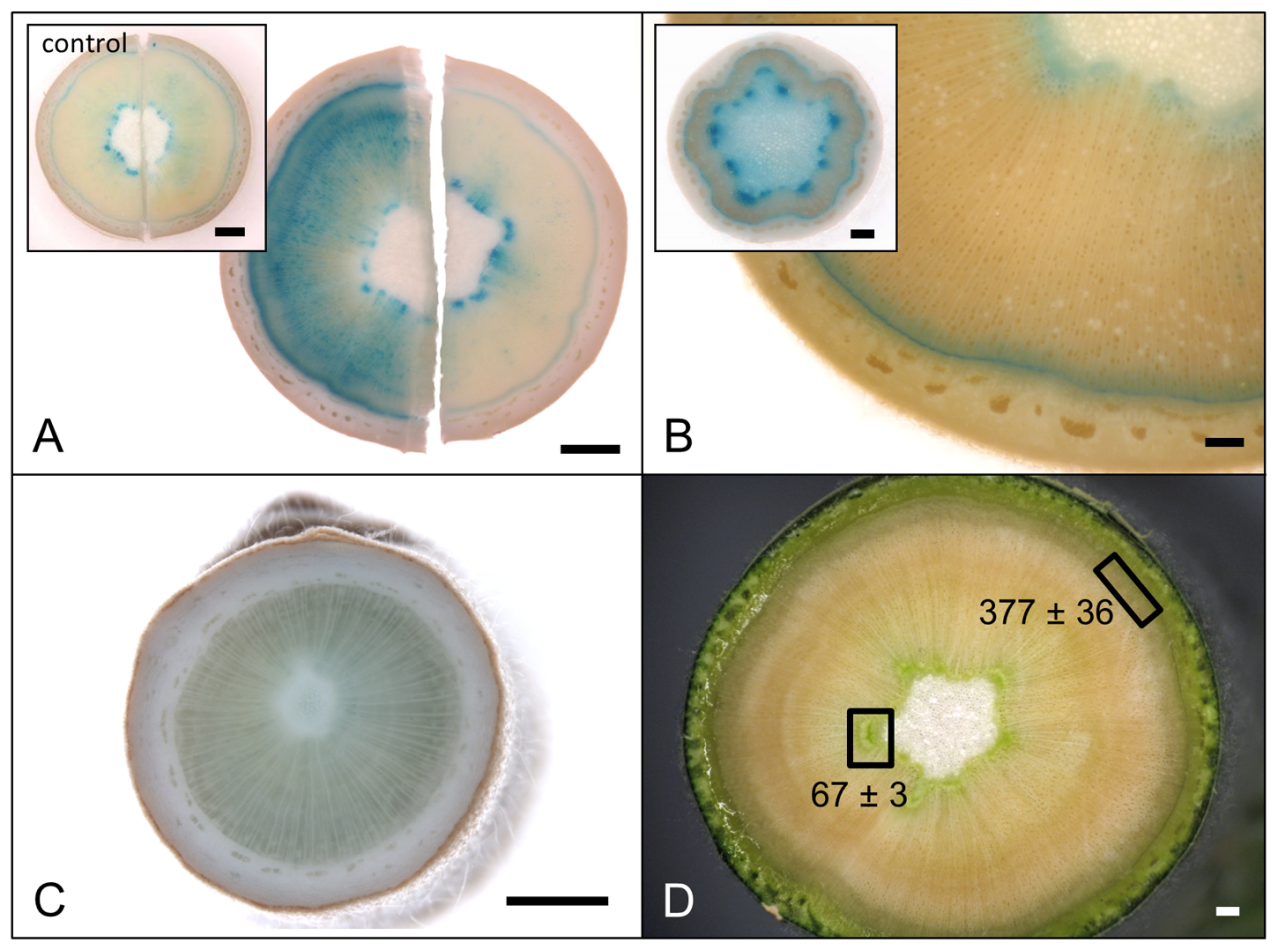
Endogenous auxin response in PtaDR5 lines and IAA quantification suggest two routes for basipetal transport. Ringing stems with NPA (50 mM in lanolin) caused an increase in signal above (a, left) the site of application; signal below the site of application (a, right) was comparable to control (inset; above mock-treatment shown on left, below on right). GUS expression in both the cambial zone and primary xylem parenchyma (PXP) continued throughout active growth, with the latter remaining up to 100 nodes beneath the apex, or as long as leaves remained firmly attached to the stem (b; stems 90 and 20[inset] nodes beneath the apex). Dormant stems lacking leaves showed no GUS expression (c). Concentrations of free IAA (ng IAA g^−1^ fresh tissue) in mature PXP and developing secondary xylem as quantified by GCMS support a role of PXP in IAA transport and/or signaling (d). In contrast, no free IAA was reliably detected in mature secondary xylem (i.e., the region between developing xylem and primary xylem; data not shown). Scale bars represent 1 mm.

All 14 PtaDR5 lines were auxin-responsive as indicated by exogenous IAA application (PtaDR5-2 shown in Figure 2) although the strength of the response varied. In the absence of exogenous IAA GUS expression was consistently found in axillary meristems, the cambial zone, poles of primary xylem parenchyma (PXP) around the outer margin of the pith, and both primary and lateral root tips (Figure 2a−c). Ringing stems with 50 mM NPA in lanolin induced strong GUS expression in the cambial zone within and immediately above the point of application; staining below the point of application was comparable to that of controls (Figure 3a). All lines showed similar endogenous expression patterns and three were selected for more detailed examination. GUS expression in both the cambial zone and PXP continued as long as leaves remained firmly attached to the stem (e.g., up to about 100−120 nodes below the apex in well-watered, greenhouse-grown trees), although the signal weakened in the oldest regions of the stem (Figure 3b; stems about 35 [inset] and 90 nodes below the apex ). GUS expression was lacking entirely in dormant stems from which all leaves had abscised (Figure 3c), as well as from actively growing stems below the zone of leaf abscission (data not shown).

Because of the pronounced and consistent GUS expression in PXP poles, tissue was collected from mature stems (internodes about 40−80 nodes beneath the apex) for IAA extraction from the region outside the pith and compared with developing secondary xylem. The amount of free IAA in developing xylem (377±36 ng g^−1^ fresh tissue) was more than five times that of primary xylem (67±3 ng g^−1^), but levels detected in primary xylem were consistent and measurable (Figure 3d). In contrast, no free IAA was reliably detected in mature secondary xylem in the same region of the stem.

### Auxin transport assays

**Figure 4:**
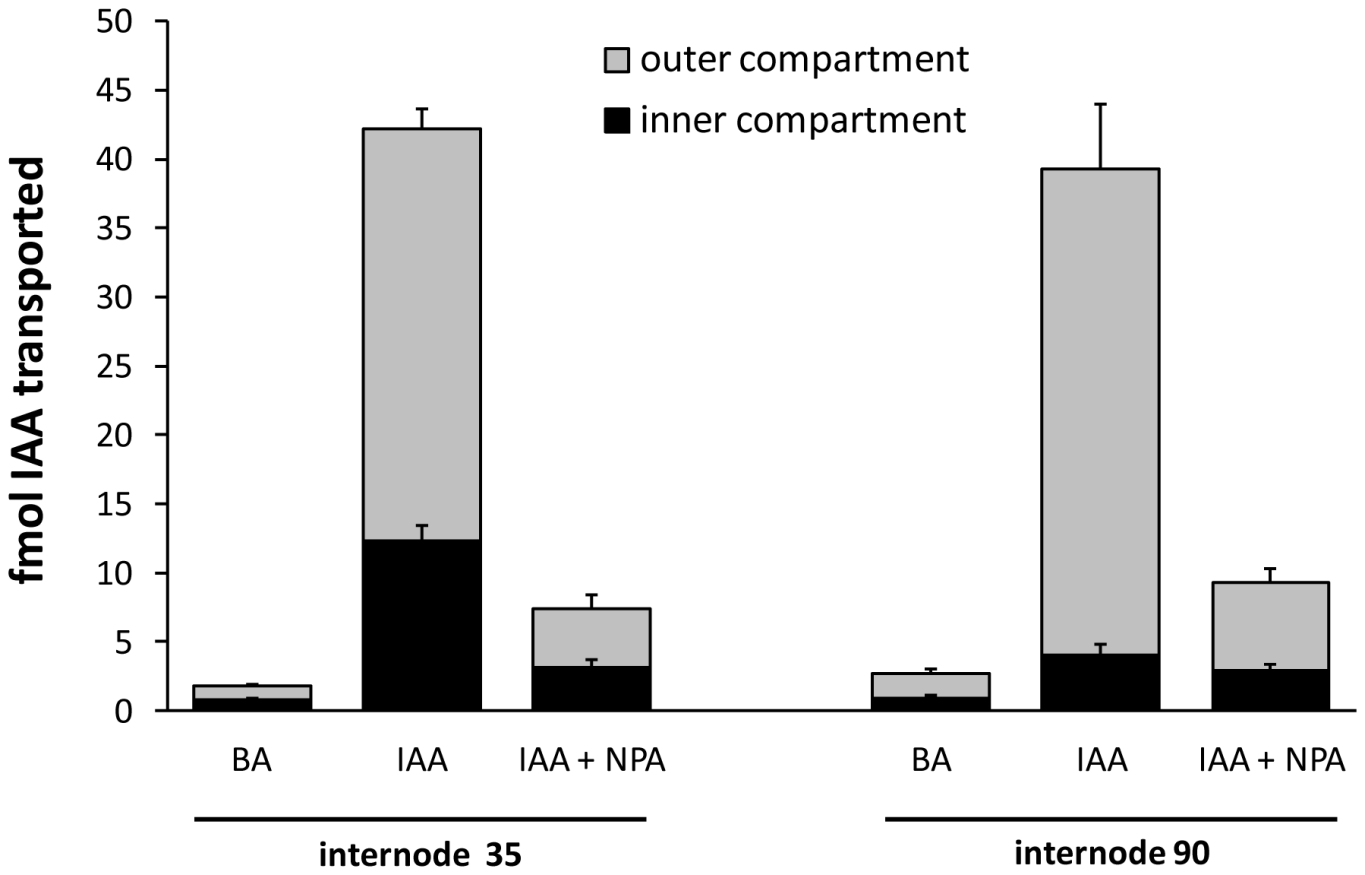
Basipetal movement of ^3^H-IAA and ^3^H-BA through *Populus* stem segments at two vertical positions. Internode 35 (i.e., stem segments approximately 35 nodes beneath the shoot apex) was mid-crown with leaves that had reached full expansion. Internode 90 was near the base of the stem with small leaves that remained firmly attached. Radiolabeled compounds were supplied in agar (1.5%. at 100 nM concentrations) to the entire apical end and collected from inner and outer compartments at the basal end. The polar transport inhibitor NPA (10 µM) reduced basipetal movement of ^3^H-IAA through the outer compartment at both stem positions, and through the inner compartment around 35, but had no effect on movement through the inner compartment around internode 90 (n  =  5 trees, with 2−3 technical replicates per tree per treatment). Refer to Figure 3b for GUS expression at comparable stages of development.

**Figure 5:**
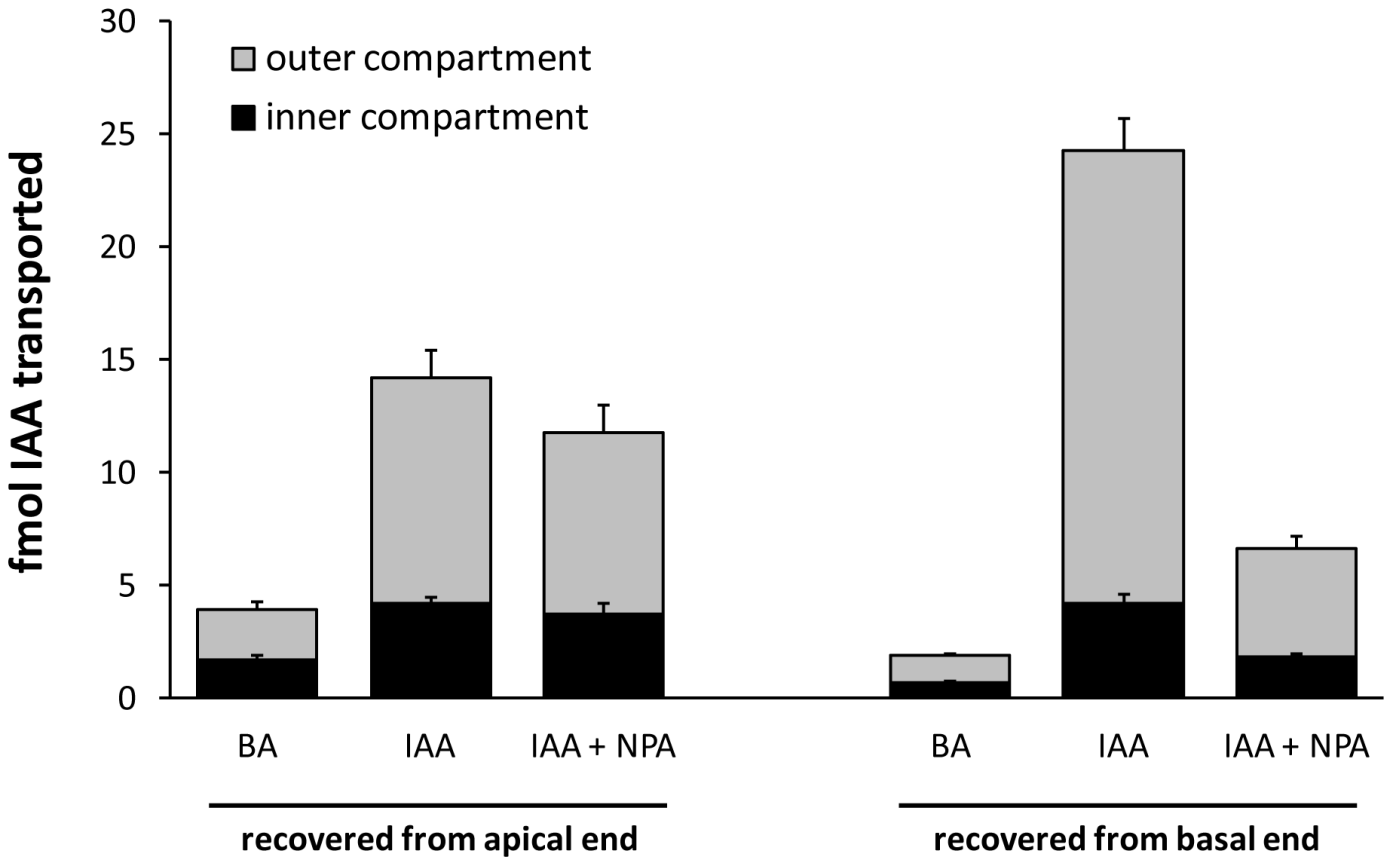
Movement of ^3^H-IAA and ^3^H-BA from a cut petiole. ^3^H-IAA and ^3^H-BA were applied to cut petioles and transported to the inner and outer vascular compartments of the apical and basal ends. ^3^H-IAA traveled significantly faster than ^3^H-BA, suggesting that its movement was facilitated. The PAT inhibitor NPA had little to no effect on apical movement, whereas basipetal movement was significantly reduced, most notably in the outer compartment (n  =  12 [BA and IAA] or 6 trees [IAA + NPA], with 3 technical replicates per tree per treatment collected between the 40th and 60th internode from the apex).

In order to determine whether polar transport of auxin could occur through both the PXP and cambial regions, a series of transport assays were conducted in which the central and outer cylinders were separated by a barrier at one or both ends of an internodal stem segment (Figure 1). Radiolabeled auxin (^3^H-IAA) applied to the entire apical cut surface was recovered from the basal end in both the outer compartment (includes cambial region) and inner compartment (includes primary xylem). At 35 internodes beneath the apex, where leaves were fully expanded, nearly 30% of the auxin transported was recovered in the inner compartment and NPA reduced transport by about 75% and 85% in the inner and outer compartments, respectively (Figure 4). Total stem IAA transport was similar at 90 internodes beneath the apex, where leaves were still firmly attached but about 10 internodes distal to the start of senescence and abscission. Here recovery from the inner compartment was reduced to 10% of the total auxin transported and was unaffected by NPA (p-value  =  0.2, one-tailed paired t-test), whereas NPA reduced recovery from the outer compartment by 83% (p-value < 0.01, one-tailed paired t-test), similar to that at internode 35. When ^3^H-IAA was supplied to a cut petiole of a fully expanded mature leaf, over 60% of the transported auxin was recovered from the basal end, and over 80% of that pool was in the outer compartment (Figure 5). Basipetal transport through inner and outer compartments was reduced by NPA by 63% and 76%, respectively (p-value < 0.01 in both cases; one-tailed paired t-test). In contrast, NPA had no effect on transport toward the apical end in either compartment.

**Figure 6:**
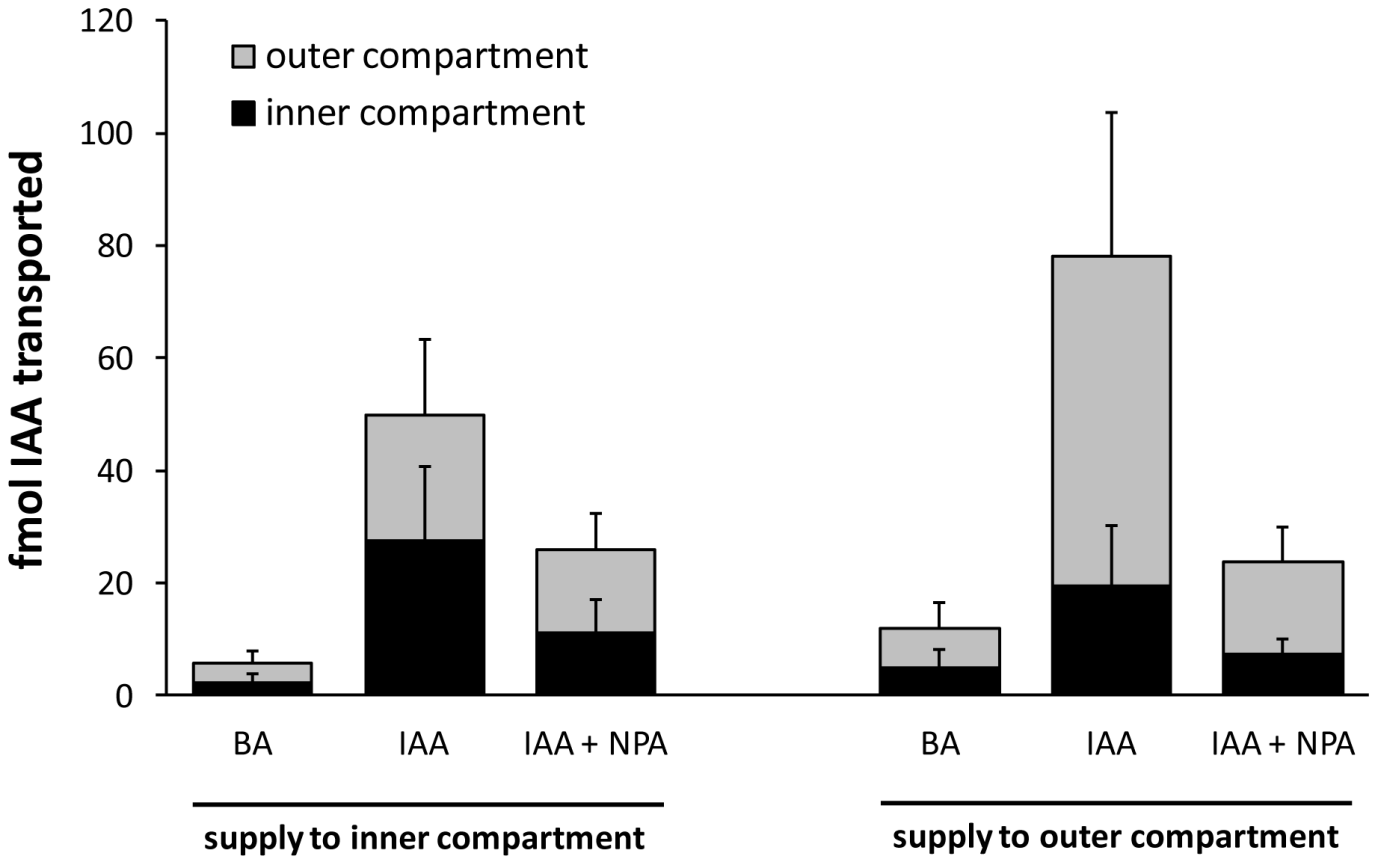
Basipetal movement of ^3^H-IAA and ^3^H-BA within and between inner and outer vascular compartments. Radiolabeled compounds were supplied in agar to one of the two compartments at the apical end; the other compartment received a control agar. Recovery of radiolabel from the basal end in the non-delivery compartment suggests a radial route of exchange. The polar transport inhibitor NPA reduced basipetal movement of ^3^H-IAA applied to and recovered from the outer compartment, but had little to no effect on recovery from the non-supply compartment (n  =  3 trees, with 3 technical replicates per tree per treatment collected between the 60th and 80th internode from the apex).

When ^3^H-IAA was supplied to one of the two compartments (either inner or outer) at the apical end it was recovered from both compartments at the basal end (Figure 6), indicating that exchange was possible in both directions. The addition of NPA reduced direct transport through the outer compartment (i.e., ^3^H-IAA supply to and recovery from the outer compartment; Figure 6) at a level that was marginally significant (p-value  =  0.08; one-tailed paired t-test comparing IAA and IAA + NPA movement through the outer compartment). Direct transport through the inner compartment was only slightly reduced by NPA (p-value  =  0.1; one-tailed paired t-test as above). No significant reduction in recovery from the non-supply compartment was observed with the addition of NPA (p-values  =  0.2 for both inner supply/outer recovery and outer supply/inner recovery compartment combinations). Radial transport of ^3^H-IAA from the perimeter of the pith to the developing xylem adjacent to the cambial zone was also slightly but significantly reduced by the addition of NPA or quercetin, with nearly twice as much ^3^H-IAA transported as ^3^H-BA (p-values ≤ 0.04 in all cases; Table 1)

**Table 1:** Radial transport of IAA ± two polar auxin transport inhibitors compared to a diffusion control.

	^3^H-IAA	^3^H-IAA + NPA	^3^H-IAA + QUE	^3^H-BA
fmol IAA (mean ± se)	9.9±1.2	8.7±0.9	8.3±0.7	6.4±0.7
p-value^†^	N/A	0.03	0.04	0.003

Radiolabeled IAA and BA (benzoic acid, diffusion control) were supplied in lanolin at 100 nM concentrations via a pith cavity to 4-month-old stems of *Populus tremula* x *alba*. PAT inhibitors N-1-naphthylphthalamic acid (NPA) and quercetin (QUE) were added at 10 µM. Radioactivity was collected from developing xylem strips after an 18 hour incubation. Two-tailed paired t-tests^†^ comparing transport to that of IAA alone indicate a highly significant increase in IAA transport relative to BA, and a small but significant decrease in IAA transport when NPA or QUE were added to the lanolin. Note that the pairing of segments within each tree (i.e., treatment replication was nested within tree) is not reflected in the overall mean but is accounted for with paired t-tests.

### Transition from primary to secondary growth in PtaDR5 lines

**Figure 7:**
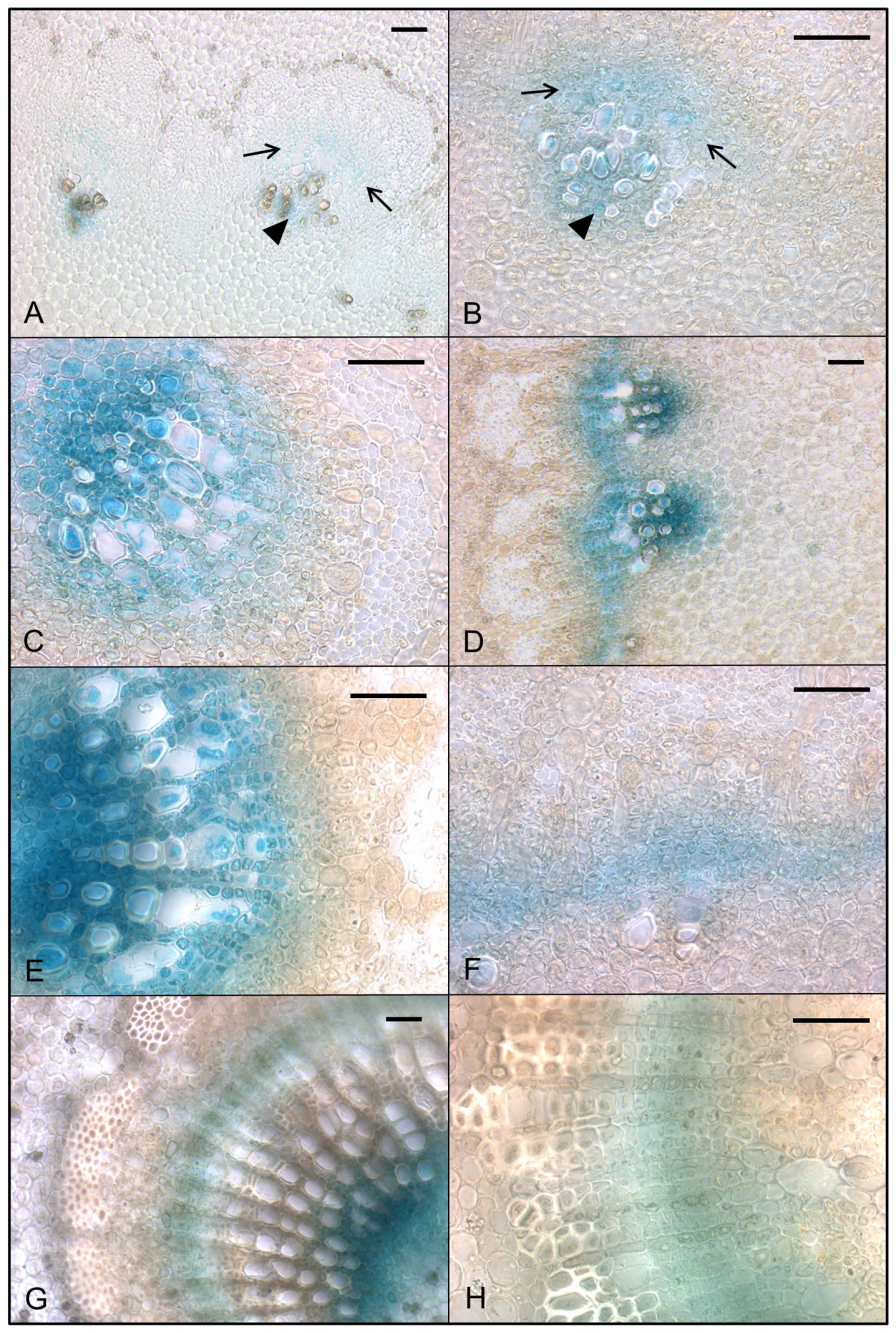
GUS expression in PtaDR5 lines during the transition from primary to secondary growth. The shoot apex was defined as the tight cluster of developing leaves above the first internode that could be clearly identified with the naked eye. This internode was defined as the first internode beneath the apex and was subtended by the first node/leaf beneath the apex. Subsequent nodes/internodes were numbered accordingly; see text for details. GUS expression in the stem at the shoot apex (a, b) was restricted to PXP (arrowheads) associated with mature and developing protoxylem and the opposing strips of procambium (bounded by arrows). The first evidence of GUS expression tangentially linking isolated poles of primary xylem (c) occurred in the third internode beneath the apex (d). Radially organized files of procambium were first clearly seen in the fifth internode beneath the apex opposite the most developed PXP poles (e), but regions between these poles in the same internode lacked radial organization; here GUS expression was found in undifferentiated cells linking the earliest developing protoxylem (f). A continuous cambium with clear radial files producing both secondary xylem and phloem was well-established in the seventh internode beneath the apex (g, h). Here the development of secondary xylem has separated regions of GUS expression in PXP and the cambial zone (g). Scale bar represents 50 µm.

Just beneath the shoot apex (defined here as the first internode that could be clearly distinguished with the unaided eye), GUS expression was restricted to isolated poles of PXP associated with the first-forming protoxylem and the opposing narrow strip of procambium (Figure 7a and b, arrowheads and arrows, respectively). Procambium associated with the most developed bundles showed signs of radial organization (Figure 7a, region between arrows) but in many this organization was lacking (Figure 7b). The first evidence of GUS expression linking vascular bundles circumferentially was found in the third internode beneath the apex (Figure 7d). Here there was clear radial organization in the procambium opposite PXP in many bundles (Figure 7c), and evidence of GUS expression in the protoplasts of rapidly expanding (and as a consequence, poorly preserved) proto- and metaxylem vessels (Figure 7c and e). An uninterrupted ring of GUS expression was generally not observed until the fifth internode beneath the apex, where radial organization of the procambium was pronounced in bundles (Figure 7e) but still lacking in between them (Figure 7f). By the seventh internode beneath the apex a fully unified ring of GUS expression could be found overlaying a radially organized vascular cambium (Figure 7g and h) producing secondary xylem and phloem to the adaxial and abaxial sides, respectively.

## Discussion

### PtaDR5 and transport assays suggest two separate but linked routes of basipetal PAT in developing *Populus* stems

The pattern of GUS expression in PtaDR5 lines is in agreement with a large body of evidence for basipetal PAT through the cambial zone, but it also suggests that strands of primary xylem parenchyma (PXP) lining the pith could function in auxin transport for as long as the leaves to which they lead remain attached to the stem. That PXP may serve as a route for PAT is not surprising given that AtPIN1 was first localized to the basal membranes of xylem-associated parenchyma in the *Arabidopsis* inflorescence axis [57], but here we show that strands of PXP in *Populus* remain competent to respond to and transport auxin long after the differentiation of primary vasculature is complete. Evidence for basipetal PAT through PXP in *Populus* stems with substantial secondary growth includes the fact that recovery of ^3^H-IAA from an inner compartment enclosing the PXP was more than 10x that of ^3^H-BA and was reduced by nearly 75% when applied with 10 µM NPA (Figure 4). This was observed consistently in stems about 35 internodes down from the apex where leaves were fully expanded. Near the base of the stem where leaves were much smaller (leaf size at full expansion increases dramatically in early development) and approaching a zone of leaf abscission, basipetal transport of ^3^H-IAA was reduced to just 4x that of ^3^H-BA and was no longer NPA-sensitive (Figure 4), suggesting a cessation of polar transport as leaves senesce. Indeed, endogenous GUS expression in PtaDR5 lines was never observed in PXP of dormant stems that had dropped their leaves (Figure 3c), nor in PXP of actively growing stems beneath the zone of leaf abscission. Finally, although the much larger pith parenchyma cells were included in the same compartment as PXP, in mature PtaDR5 stems pith cells showed no GUS expression (no endogenous expression [e.g., Figure 3b] nor any in response to exogenous IAA application except just beneath the shoot apex), so they are unlikely to have contributed significantly to the transport described here.

In order to test for transport through the inner compartment of several-month-old stems it was necessary to work with excised stem segments. Although less invasive techniques are of course preferred, several observations suggest that these measurements are valid for relative comparisons and that transport through PXP occurs in intact woody plants. First, although phloem transport is certain to be disrupted, the physiology of excised woody tissue is typically robust for 48 hours or more after excision, including rates of parenchyma respiration and water transport capacity [58], [59]. Second, tissue polarity was maintained in our segments after excision from the stem such that the ratio of basipetal to apical transport was the same regardless of whether segments were incubated apical or basal side up (data not shown). Third, our use of a relatively low concentration of ^3^H-IAA should minimize any increase in PAT capacity in response to exogenous auxin. In younger stems where physical separation of the two compartments is not feasible, localizing PAT could be achieved through autoradiography. Although not quantitative, autoradiographic images from both *Pisum* [60] and *Fagus* [61] suggest PAT through PXP during active growth in addition to through the cambial zone. Recent synthesis of 3-indolyl[1-^11^C] acetic acid [62] and its visualization with positron autoradiography may provide new avenues for localization of PAT in stems *in vivo*.

In stems with secondary growth the inclusion of a BA control is particularly important as there are several potential routes of auxin movement. Although the separation of inner and outer compartments with a physical barrier is a useful technique, it is important to note that both compartments include secondary xylem, such that ^3^H-IAA and ^3^H-BA are effectively applied to the water-filled vessel elements and living ray parenchyma in addition to the cambial zone and PXP. Although diffusion through xylem water is possible, transport through ray parenchyma is likely to be a far more important route. We found that when either radiolabeled compound was supplied to just one compartment (i.e., inner versus outer) substantial quantities were recovered from the other compartment, a process that implies transport through the rays. It is not known whether this pathway was dominated by introduction into the PXP and subsequent transfer to the rays, or by direct introduction to the rays themselves. In either case, direct basipetal transport through the rays is unlikely to occur as rays do not extend longitudinally through an internode and transfer between rays within the secondary xylem requires axial parenchyma, which is scarce in *Populus*. Ray parenchyma in *Populus* consists of radially elongate cells between 100 µm and 150 µm long that are symplasmically connected via plasmodesmata [63], [64], running from the margin of the pith through the cambium and into the secondary phloem. Several rays link each pole of PXP to the cambial zone but are otherwise isolated from each other. Although rays form a route of exchange between secondary xylem and phloem, the regulation and mechanism of radial transport in woody plants is poorly understood (see [65−68] for reviews).

Our estimate of outward radial (i.e., centrifugal) auxin transport is admittedly crude but it points to two important observations. First, radial transport of ^3^H-IAA was about 1.5x greater than that of ^3^H-BA. This could simply be a function of a retrieval or uptake mechanism in parenchyma cells for IAA but not BA. We have found that several members of the three gene families known to encode auxin transport proteins are expressed in the ray parenchyma cells of mature secondary xylem in *Populus*, including PIN1, AUX2, ABCB1 an ABCB7 [30]. Second, radial transport of ^3^H-IAA was significantly, albeit only slightly (by about 10−15%), reduced by inclusion of 10 µM NPA or 10 µM quercetin (Table 1). That these PAT inhibitors reduced transport at all is surprising given that radial transport is expected to be symplasmic and should not involve membrane transport. It is not known whether the observed minor reduction in transport reflects inhibition of PAT in axial PXP prior to transfer to the rays or a reduction in radial transport itself, but the former would be expected to contribute. In either case, given that no free IAA could be detected reliably in mature secondary xylem (whereas measurable quantities were found in tissue enriched in PXP, and greater quantities in the cambial zone; Figure 3d), it seems unlikely that rays provide a major route for free IAA transport in stems. What our measurements demonstrate is simply that transfer of IAA from PXP to the cambial zone can occur, and that this transfer appears facilitated in some way. Given our limited understanding of radial transport and ray cell metabolism (e.g., its direction may be centripetal or centrifugal depending on phenological timing and solute concentrations [63], [66], ), it is not known whether rays might transport conjugated forms of IAA or to what extent they contribute to whole stem IAA metabolism and transport. Metabolic processing of proteins and carbohydrates is well documented in rays cells [65] but to our knowledge their potential to process or transport hormones has never been documented. Although there are no good published estimates of transport velocity due to the complexity of the system, symplasmic transport is expected to greatly exceed intercellular PAT. It is important to note however that because our pith-filling technique required the use of lanolin as a delivery method instead of the agar used in all other assays reported here (see Methods), the quantities of ^3^H-IAA and ^3^H-BA delivered via radial transport over a given number of hours are not comparable with those reported for axial transport.

### Strands of PXP in the stem are functionally connected to the leaves

**Figure 8:**
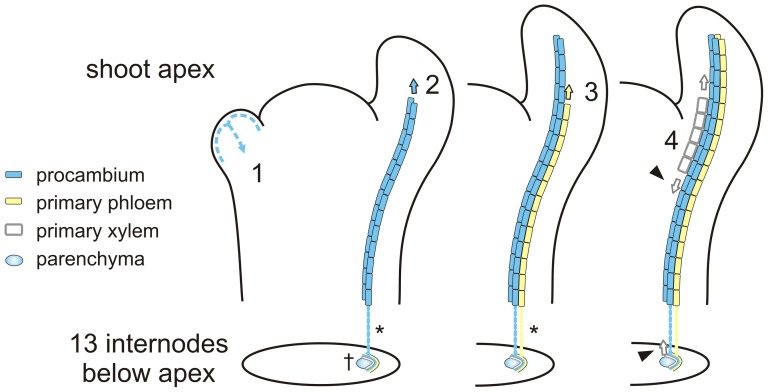
Vascular connections between developing leaf primordia and primary vascular bundles in the stem below are a function of phyllotaxis. Current molecular work suggests that auxin is first transported up through the epidermis and then channeled (i.e., canalized) down through the center of emerging leaf primordia (1), and that this process precedes the differentiation of procambium (2). Classical development work has shown repeatedly that procambial differentiation procedes acropetally into the expanding primordium, continuous with the procambial strands below (*), although when and how this process initiates is not known. Similarly, classical work has shown that primary phloem is the first vascular tissue to differentiate from procambium (3), and that its development is acropetal and continuous with the primary phloem below (*). In contrast, primary xylem differentiation is discontinuous (4), lags behind primary phloem, and proceeds both acropetally and basipetally (arrowheads). The work described here suggests that strands of parenchyma (†) form a second basipetal route for PAT. Although the developmental role for this tissue is not known, it may serve as a guide for developing primary xylem, which appears bounded on both abaxial and adaxial sides by tissue involved in PAT (procambium and PXP, respectively).

In *Populus*, three strands of PXP traverse the secondary xylem, depart the stem and enter the petiole in a compound leaf trace (personal observations with dye injections in *Populus tremula* x *alba*; in agreement with [71]). Although the functional significance of auxin transport through these strands of PXP is not known, they could provide an important route of communication between mature leaves and the stem. During development and/or following wounding, strands of PXP could also serve as an auxin sink to guide the basipetal transport of auxin necessary to link new growth to existing vascular bundles. The three-dimensional structure and chronological sequence of secondary vascular development is extremely complex, but a series of papers by P. Larson and colleagues painstakingly document the development of primary and secondary growth as it relates to phyllotaxy in *Populus deltoides* [71−78]. This development appears identical to that of *Populus tremula* x *alba* by our observations. Both share a 5/13 phyllotactic arrangement, meaning that there are 13 internodes between two leaves in direct axial alignment and that within this span leaves circle the stem five times. Thus, a primordium at the apex shares a direct vascular connection with the expanding leaf 13 nodes below owing to the pattern of procambial development in *Populus* (Figure 8). Our work suggests that this connection remains throughout the life of the leaf in the form of living PXP, long after the procambium has developed into a mature vascular cambium. For young developing leaves exporting free IAA the procambium/cambium continuum is likely the predominant basipetal path (Figure 8), but the timing and nature of the partitioning of procambial versus PXP tissues, both of which appear capable of basipetal PAT, remains an open question. Non-invasive, *in vivo* techniques are needed to determine whether both acropetal and basipetal transport are possible through these strands. Finally, although mature leaves are unlikely to be a direct source of free IAA, there is evidence that IAA exported in the phloem from mature leaves can enter the PAT pathway [39], [40], [60]. The anastomosing network of PXP linking leaves of different ages via the interior of the stem, as well as the radial network linking PXP to the cambium and secondary tissues, must now be also considered as a potential pathway.

### PtaDR5 lines suggest a role for basipetal IAA canalization in both primary and secondary xylem development

A young *Populus* stem is particularly complex because it includes a ring of vascular bundles that are all at different stages of development [35], where the extent to which each bundle has matured is a function of the age of the leaf to which it is connected [71], [72]. Even the three bundles that will enter a single leaf vary in developmental stage, with the central trace being the most mature. Similarly, although the criteria researchers use to distinguish primary from secondary growth varies, the onset of secondary growth is not simultaneous within a given stem section (shown here, in agreement with [77]). Here we show an auxin response in developing PtaDR5 stems beneath the shoot apex, first in isolated strands of procambium and the associated developing protoxylem. Although the rapidly expanding protoxylem elements are difficult to preserve prior to significant cell wall formation, the protoplasts of these cells show strong GUS expression prior to programmed cell death (which concludes maturation; Figure 7b, c and d) suggesting possible canalization of IAA through the axially aligned vessel elements. Several nodes down from the apex GUS expression in both the PXP and procambium/differentiating xylem intensifies as the distance between these two regions increases, and GUS expression first appears in the undifferentiated parenchyma between bundles (Figure 7d and f). As in leaf development [2], this suggests that an auxin response precedes the appearance of an organized (pro)cambium in the stem (Figure 8). Exactly how this continuous ring of GUS expressing, auxin-responding tissue is formed is an open question, but it is clearly continuous with a true vascular cambium below. The most likely source of auxin eliciting this response is the tight spiral of rapidly developing leaves above, but it is not known to what extent PIN-directed basipetal and/or lateral transport maintains the boundaries of this zone. Finally, recent work on the role of brassinosteroids and auxin transport in vascular development [79] has identified similar 'auxin maxima' associated with developing vascular bundles in the *Arabidopsis* inflorescence axis, but the authors do not distinguish between developing xylem elements and parenchyma. In fact, their images show DR5-driven expression in *Arabidopsis* axes with similar localization to that shown here, with signal in distinct clusters of parenchyma adjacent to expanding vessel elements, as well as the opposing procambium.

In order to interpret the patterns of auxin response and transport in woody stem development shown here it is important to make a distinction between the role of PAT in procambium initiation versus its role in xylem differentiation. Molecular studies linking PAT to vascular differentiation have focused on the origin of procambium in leaves and show beautifully how the PIN efflux carriers are localized to channel (i.e., 'canalize') auxin through narrow files of cells, thereby establishing the basic layout of procambium and hence leaf venation [34]. In contrast, the focus of classical work has been on PAT and xylem differentiation, in part because experimental manipulations involved wounding and the subsequent regeneration of xylem directly from parenchyma (e.g., [80−83]), but also because the thick cell walls of xylem elements made them easy to visualize. More recently a picture has emerged from studies of root vascular development linking the two processes in which cytokinins, delivered to the procambium by the phloem, indirectly promotes PIN localization to the lateral membranes of procambial cells such that a bisymmetric pattern of auxin maxima forms and leads to protoxylem specification [84], [85]. These findings are exciting in light of two key observations from classical development studies: in the shoot apex, both procambium and (later) phloem differentiate acropetally and in continuity with strands below, while xylem differentiation is discontinuous (i.e., it initiates in at least two positions), can proceed both acropetally and basipetally, and always lags behind phloem differentiation (Figure 8; [11], [12], [71], [72], [86], [87]). It is possible that phloem-derived signals play a role in shaping this ring of auxin-responding tissue as well. Further down the stem, xylem differentiation begins adjacent to existing vasculature and proceeds acropetally (Figure 8). It is tempting to speculate that the appearance of protoxylem differentiating within (and centripetal to) this ring of undifferentiated tissue showing GUS expression (e.g., Figure 7f), but where PXP is lacking, represents this acropetal xylem differentiation. Detailed anatomical studies with better spatial resolution of the auxin response are needed for a more complete understanding of this process.

A fully unified vascular cambium with clear radial files of initials and derivatives appears between the fifth and seventh internode beneath the apex. Here GUS expression is more sharply delineated on the side of the phloem, with apparent channeling of auxin down through the differentiating xylem elements as seen in primary xylem. This is in keeping with microscale measurements of IAA concentration through the cambial zone that show a more gradual decline on the side of the developing xylem [23−25]. It will be interesting to see whether phloem-derived cytokinins are involved in maintaining the shape of this basipetal stream of auxin, as might be suggested by the sharper phloem-side boundary. One final observation provides tantalizing evidence that the phloem may play a role in shaping the transition from primary to secondary growth: the region of the stem where this transition takes place in *Populus* (i.e., the 5th, 6th or 7th internode beneath the apex) coincides with the age at which leaves transition from being sinks to sources of photosynthate [88], [89].

## Conclusions

In summary, the multiple routes of PAT in *Populus* stems described here together with an increasing appreciation of the role of phloem transport and the potential for local sites of IAA biosynthesis suggest that the dynamics of auxin transport in woody stems are more complex than once thought. Although text book models of the transition from primary to secondary growth usually depict an abrupt shift from separate vascular bundles to continuous rings of xylem and phloem, the unification of these bundles is quite gradual. More importantly, these bundles anastomose throughout the stem and contain strands of parenchyma that function in PAT for as long as the leaves to which they are connected remain alive. Ray cells in turn provide a symplasmic route of exchange between these strands of primary xylem parenchyma and the developing vascular cambium. Given the longevity of ray parenchyma and their capacity for relatively rapid radial transport, future research should focus on the potential of these cells to synthesize, metabolize and transport auxin as well as other key plant growth regulators.
